# Isoniazid Toxicity among an Older Veteran Population: A Retrospective Cohort Study

**DOI:** 10.1155/2013/549473

**Published:** 2013-01-10

**Authors:** Christopher Vinnard, Anand Gopal, Darren R. Linkin, Joel Maslow

**Affiliations:** ^1^Division of Infectious Diseases and HIV Medicine, College of Medicine, Drexel University, 245 N. 15th Street MS 461, New College Building 6314, Philadelphia, PA 19102, USA; ^2^College of Arts and Sciences, University of Pennsylvania, Philadelphia, PA 19104, USA; ^3^Division of Infectious Diseases, Department of Medicine, Perelman School of Medicine, University of Pennsylvania, Philadelphia, PA 19104, USA; ^4^Department of Medicine, Philadelphia VA Medical Center, Philadelphia, PA 19104, USA; ^5^Center for Clinical Epidemiology and Biostatistics, Perelman School of Medicine, University of Pennsylvania, Philadelphia, PA 19104, USA; ^6^Division of Infectious Diseases, Morristown Medical Center, Morristown, NJ 07962, USA

## Abstract

*Background*: our objective was to determine the incidence of toxicity among veterans initiating isoniazid therapy for latent tuberculosis infection (LTBI) and determine whether advancing age was a risk factor for toxicity. *Methods*: we performed a retrospective cohort study among all adults initiating isoniazid treatment for LTBI at a Veterans Medical Center from 1999 to 2005. We collected data on patient demographics, co-morbidities, site of initiation, and treatment outcome. *Results*: 219 patients initiated isoniazid therapy for LTBI during the period of observation, and the completion of therapy was confirmed in 100 patients (46%). Among 18/219 patients (8%) that discontinued therapy due to a documented suspected toxicity, the median time to onset was 3 months (IQR 1–5 months). In an adjusted Cox regression model, there was no association between discontinuation due to suspected toxicity and advancing age (HR 1.03, 95% CI 0.99, 1.07). In contrast, hepatitis C infection was a significant predictor of cessation due to toxicity in the adjusted analysis (HR 3.03, 95% CI 1.08, 8.52). *Conclusions*: cessation of isoniazid therapy due to suspected toxicity was infrequently observed among a veteran population and was not associated with advancing age. Alternative LTBI treatment approaches should be further examined in the veteran population.

## 1. Introduction

According to World Health Organization estimates, one-third of the world's population is latently infected with *M. tuberculosis*, and 10% of immunocompetent individuals will progress from latent to active tuberculosis infection within their lifetimes [1]. Detection and treatment of latent tuberculosis infection (LTBI) remain a cornerstone of the strategy to reduce the incidence of active tuberculosis in the United States. Daily isoniazid therapy for six to 12 months has been shown to significantly reduce the risk of progression from latent to active tuberculosis infection [2].

In the 1970's, several cases of fatal hepatotoxicity during isoniazid therapy for LTBI raised concerns regarding widespread isoniazid use and led to a reconsideration of its safety in older adults [3]. Subsequent studies of isoniazid toxicity have compared the risk of hepatotoxicity between adults less than and greater than 35 years of age [4–10], and a meta-analysis of these studies demonstrated a small but statistically significant increased risk of hepatotoxicity among adults greater than age 35 [11]. Because of these concerns, providers may be more reluctant to initiate isoniazid therapy in older patients with LTBI, particularly in the presence of comorbid illnesses [12].

Less is known about the relationship of advancing age and isoniazid toxicity among adults in later decades of life, particularly when used in clinical practice outside of tuberculosis control programs or public health clinics. Furthermore, although isoniazid treatment is recommended for use in long-term care facilities as a component of infection control programs [13], prior epidemiologic studies of isoniazid toxicity have been limited to outpatient settings. 

Veterans are a unique population for LTBI-screening efforts, given their additional risk of tuberculosis exposure related to service deployments [14]. We sought to describe the incidence of toxicities among veterans initiating daily isoniazid treatment of LTBI and determine whether advancing age was associated with isoniazid discontinuation due to suspected toxicity.

## 2. Methods

We performed a retrospective cohort study of LTBI treatment with isoniazid at a single medical center. The Philadelphia Veterans Affairs (VA) Medical Center is a federal health care facility comprising an outpatient clinical facility, a 145-bed acute care hospital, and an adjoining 240-bed long-term care facility. We queried pharmacy records to identify all individuals prescribed isoniazid between January 1, 2000, and December 31, 2005. Among these patients, we limited study inclusion to individuals receiving isoniazid alone for the treatment of LTBI. 

We reviewed clinical charts to extract demographic and comorbidity data, including HIV infection, hepatitis C infection, past or current alcohol abuse, and past or current intravenous drug use (IDU). Laboratory records were reviewed for baseline serum transaminase levels (defined as levels obtained within 6 months prior to initiation of isoniazid therapy), along with peak serum transaminase levels during treatment. 

We classified treatment outcomes as completion of therapy, discontinuation due to a suspected toxicity, or discontinuation for unknown reasons (if completion could not be documented either by review of the clinical chart or the pharmacy records). We calculated the 95% confidence interval (CI) for the incidence of toxicities using the binomial test. We performed Cox regression analysis to determine the adjusted association of advancing age with treatment discontinuation due to suspected toxicity, which allowed us to account for differences in observation time. Stata v11.0 (College Station, TX, USA) was used for statistical analysis, and significance was declared for *P* values less than 0.05.

## 3. Results

Overall, 219 veterans initiated isoniazid therapy for LTBI, with a median age of 53 years (range 22–90). There were 18 patients (8%) with therapy discontinued due to suspected toxicity, 100 patients (46%) with successful completion of therapy, and 101 patients (46%) with therapy discontinued for unknown reasons. Isoniazid completion rates were higher among residents of the long-term care facility compared with nonresidents (64% versus 44%, *P* = 0.07), with similar rates of discontinuation for suspected toxicity between these two groups (9% versus 8%, *P* = 0.88). Among patients initiating treatment in the outpatient setting, there was no difference in completion rates for patients initiating treatment in the primary care clinics (40%) compared with the infectious disease clinic (49%, *P* = 0.22).

Baseline alanine transaminase (ALT) levels were obtained in 184 of 219 patients (84%). During isoniazid therapy, followup ALT levels were obtained in 156 of 219 patients (71%) overall, including 15 of 18 patients (83%) with subsequent cessation due to suspected toxicity (15/18, 83%) compared to 141 of 201 patients (70%) without treatment cessation due to suspected toxicity.

Discontinuation for suspected toxicity occurred in 18 patients (8%) after a median of 3 months (IQR 1–5 months). Hepatotoxicity was cited by the clinician as the reason for discontinuing therapy in 7 patients (3%, 95% CI 1–6%). Other suspected toxicities that led to early treatment cessation included 3 patients with rash (1%, 95% CI 0–4%) and 2 patients with nausea (1%, 95% CI 0–3%). Additional 5 patients had other types of clinical events attributed to isoniazid (malaise, back pain, thrombocytopenia, angioedema, and fever). 

Characteristics of patients with and without isoniazid discontinuation due to suspected toxicity are shown in Table 1. The median age of patients that completed therapy was 53.4 years (IQR 45.8–66.1), and the median age of patients that discontinued therapy due to suspected toxicity was 54.5 years (IQR 50.5–61.5). Only hepatitis C infection was associated with discontinuation due to suspected toxicity (*P* = 0.01). There were two patients (1%, 95% CI 0–3%) with biochemically confirmed hepatotoxicity that met the definition of ATS/CDC treatment guidelines [2]. One 50-year-old patient with a history of alcohol abuse had an ALT rise from a baseline of 80 to a peak of 329 after 104 days of treatment, and a second 80-year-old patient without identifiable risk factors had an ALT rise from a baseline of 57 to a peak of 438 after 27 days of treatment. In both of these instances there was complete resolution of hepatotoxicity with cessation of isoniazid therapy.

In a proportional hazards model that included age, hepatitis C infection, and a history of alcohol use, age was not associated with treatment discontinuation due to suspected toxicity (HR 1.03, 95% CI 0.99, 1.07). In contrast, the relationship between hepatitis C infection and isoniazid discontinuation due to suspected toxicity remained significant even after adjusting for age and alcohol use (HR 3.03, 95% CI 1.08, 8.52).

## 4. Discussion

Daily isoniazid therapy remains the cornerstone of a targeted testing and treatment approach for LTBI [2]. In an older cohort of veterans initiating isoniazid treatment for LTBI, we found a high rate of discontinuation due to suspected toxicity. In contrast, the rate of biochemically confirmed hepatotoxicity was much lower and similar to previous reports. We found that overall completion rates were lower than previous reports from public health clinics [15, 16]. 

Cessation of isoniazid therapy due to suspected toxicity was not associated with advancing age. Our work adds to prior studies of age-related isoniazid toxicity risk by enrolling a significant number of patients in later decades of life. The median age of participants in our study (53 years) is greater than any previous study of isoniazid therapy for LTBI. Treatment guidelines' use of 35 years of age has been called an “arbitrary” cutoff to define the risk of isoniazid-related toxicity [11], given that many other therapeutic drugs display significant changes in toxicity risk with advancing age. We found that isoniazid can be safely administered to older veterans provided that there is an appropriate clinical followup, consistent with current recommendations for the treatment of LTBI [2].

In contrast, we found that hepatitis C infection was associated with cessation of therapy due to concerns for toxicity. Our findings differ from a study of intravenous drug users in Spain that did not find a relationship between hepatitis C infection and hepatotoxicity [10]. One possible explanation for this difference is that providers in the VA were more cautious with discontinuation of therapy with minor elevations of transaminases that may not have resulted in biochemically confirmed hepatotoxicity. However, it is also possible that a biological interaction between age and hepatitis C led to our findings, given that our cohort was much older than the Spanish study (mean age 31 years). Further work is needed to characterize the complex relationship between hepatitis C and the risk of isoniazid-associated hepatotoxicity.

Interestingly, patients initiating isoniazid treatment in the long-term care facility, despite their older age and greater comorbidities, had higher rates of treatment completion compared with patients in all other settings. While isoniazid is recommended for the treatment of LTBI in long-term care facilities, there have not been specific investigations to evaluate its safety in this setting [12]. Our findings support the safety of current practices regarding LTBI screening and treatment in long-term care facilities.

Our retrospective cohort study had several limitations. Among patients lost to followup, we could not determine whether the development of side effects (such as gastrointestinal intolerance) contributed to their discontinuation of care. Furthermore, there was tremendous variability in clinical practices, with regards to testing for comorbidities (HIV or hepatitis C), number of isoniazid tablets provided per prescription, and frequency of followup visits. Our approach to ascertainment of comorbidities such as HIV or hepatiits C identified only those patients with a positive test, and the proportion of patients in the cohort without testing performed was not determined. Typical of a veteran population, women were underrepresented in this study. However, one strength of our approach was the ability to link centralized pharmacy records with clinical charts.

In summary, nine months of daily isoniazid therapy for the treatment of LTBI in an older veteran population were associated with low rates of treatment completion overall. Rates of treatment discontinuation due to suspected toxicities were similar to previous reports, and not associated with advancing age. Alternative LTBI treatment approaches should be further examined in the older veteran population.

## Figures and Tables

**Table 1:** Characteristics of veterans with and without isoniazid discontinuation due to suspected toxicity.

Characteristic	Treatment completion (%)	Discontinuation due to suspected toxicity (%)	*P* value
	(*n* = 100)	(*n* = 18)	
Age category			0.64
<35	5 (5)	0 (0)	
35–55	49 (49)	10 (56)	
56–75	35 (35)	5 (28)	
>75	11 (11)	3 (17)	
Sex			0.39
Male	98 (98)	17 (94)	
Female	2 (2)	1 (6)	
Comorbidities			
HIV	9 (9)	1 (6)	0.63
Hepatitis C	17 (17)	8 (44)	0.01
Alcohol use	33 (33)	8 (44)	0.35
IDU	11 (11)	5 (28)	0.06
Initiation setting			0.18
Outpatient			
Primary care clinic	33 (33)	7 (39)	
Infectious disease clinic	47 (47)	6 (33)	
Emergency department	3 (3)	0 (0)	
Inpatient			
Long-term care facility	14 (14)	2 (11)	
Acute care facility	3 (3)	3 (16)	
